# Exo-miRExplorer: A Comprehensive Resource for Exploring and Comparatively Analyzing Exogenous MicroRNAs

**DOI:** 10.3389/fmicb.2017.00126

**Published:** 2017-02-01

**Authors:** Ling-Ling Zheng, Kai-Wen Deng, An-Cheng Deng, Jie Wu, Jian-Hua Yang, Zhao-Rong Lun, Liang-Hu Qu

**Affiliations:** Key Laboratory of Gene Engineering of the Ministry of Education, State Key Laboratory of Biocontrol, RNA Information Center, School of Life Sciences, Sun Yat-sen UniversityGuangzhou, China

**Keywords:** exogenous, microRNA, deep-sequencing, organisms, contamination

## Abstract

MicroRNAs (miRNAs) are small regulatory RNAs that play important roles in animals, plants, and viruses. Deep-sequencing technology has been widely adopted in miRNA investigations. However, it is still a big mysterious why nearly all sequencing data contain miRNA sequences from exogenous species, called exo-miRNAs. In this study, we developed a novel platform, exo-miRExplorer, for mining and identifying exo-miRNAs from high-throughput small RNA sequencing experiments which originated from tissues and cell lines of multiple organisms. Thousands of exo-miRNAs are characterized with their expression abundance, the RNA families, original organisms and the sequencing platforms presented in exo-miRExplorer. Subsequently, we used exo-miRExplorer to perform further analysis. Comparative analysis of the exo-miRNAs between different sequencing datasets revealed significant correlation of exo-miRNAs between experiments in the same study. The plant-derived exo-miRNAs analysis provided robust evidence for non-diet source of exo-miRNAs. Virus-derived exo-miRNA analysis showed that pathogen RNAs could transfer to host cells and exist in deep-sequencing result at abundance level. In conclusion, exo-miRExplorer provides users with an integrative resource to facilitate detection and analysis of exo-miRNAs. exo-miRExplorer is available at the following URL: http://rna.sysu.edu.cn/exomiRDB/.

## Introduction

MicroRNA (miRNA) is a class of small RNA with 18–24 nt length, and widespread distribution in animals, plants, and viruses (Lee et al., 1993; Bartel, 2004). microRNA genes are first transcribed into primary miRNA transcript (pri-miRNA), and then cleaved by RNase III enzyme to generate precursor miRNA (pre-miRNA) (Lee et al., 2003). The pre-miRNAs are about 70–100 nt length and formed hairpin structure. They are further processed into a pair of small RNAs—mature miRNA and miRNA star in the cytoplasm and loaded into the Argonaute complex (Lee and Doudna, 2012). miRNAs could regulate gene expression at the post-transcriptional level through RNA interference (RNAi) mechanism (Fire et al., 1998) by pairing to the 3′ UTR of mRNA (He and Hannon, 2004). miRNAs are involved in almost all biological process in cell, especially in development and differentiation, while their abnormal expression can lead to cause cancer and other diseases (Esquela-Kerscher and Slack, 2006; Bartel, 2009; Ameres and Zamore, 2013).

Next-generation sequencing (NGS) or deep sequencing technology could rapidly retrieve almost all RNA sequences in cells, which becomes a powerful method to investigate microRNAs. Interestingly, most, if not all, sequenced datasets contain small RNA fragments from the genome of exogenous species, called exo-miRNAs (exo-miRNAs) (Jeang, 2012). These fragments were always being discarded from subsequent analysis. Recently, several studies found exogenous plant microRNAs in the blood plasma of humans and other animals by small RNA deep sequencing experiments (Wang et al., 2012; Zhang L. et al., 2012; Liang et al., 2014; Zhou et al., 2015). A representative study found 25 known plant miRNAs in Chinese healthy donors, among which miR168a and miR156a showed considerable levels of expression (Zhang L. et al., 2012). These authors further considered that these exo-miRNAs derived from food intake, and believed that these exo-miRNAs have cross-kingdom regulation functions (Zhang L. et al., 2012; Zhou et al., 2015). Later, they even found that these exo-miRNAs could cross the placental barrier to regulate the fetal gene expression (Li et al., 2015). Some researchers followed these idea to successfully detect dietary RNAs in ingesting mammals (Liang et al., 2015; Yang et al., 2015; Chin et al., 2016). Other researchers, however, disputed that these exo-miRNAs were most likely originated from environmental RNAs pollution in the process of experiments (Zhang Y. J. et al., 2012; Dickinson et al., 2013; Snow et al., 2013; Witwer et al., 2013; Lusk, 2014; Tosar et al., 2014; Witwer and Hirschi, 2014; Witwer, 2015; Bagci and Allmer, 2016), which could disturb the assessment of real gene expression and possibly affecting conclusions derived from the NGS data analysis (Tosar et al., 2014).

These studies sparked keen debate and drew much attention from both academic and commercial community (Jiang et al., 2012; Vaucheret and Chupeau, 2012; Wang et al., 2012; Witwer, 2012; Chen et al., 2013). Several controversial questions have been raised by researchers. What organisms are the origin of these exo-miRNAs? How can these exo-miRNAs get into the sequencing datasets? Could the same exo-miRNA also be re-detected in the sequencing result performed by another research group? To answer these questions, we need both a comprehensive view of exo-miRNA spectra in the whole cell, and comparative analysis of them between different studies using different sequencing methods and platforms.

To address these questions, we established a specific resource, exo-miRExplorer, to comprehensively explore the exo-miRNAs from high-throughput small RNA sequencing experiments. A total of 563 published small RNA sequencing datasets were used for exo-miRNAs classification and annotation, including the messages of expression abundance, the RNA families, original organisms, and sequencing platforms (Table S3). Subsequently, we used exo-miRExplorer to perform comparative analysis of the exo-miRNAs. In addition, we also developed a dynamic web interface to facilitate the integration, interactive evaluation, and visualization of these exo-miRNAs. Our exo-miRExplorer will facilitate scientists to effectively recognize the exo-miRNAs in the deep-sequencing experiments and reduce the potential risk from experimental contamination.

## Materials and methods

### Obtain small RNA sequencing data

A total of 563 small RNA deep-sequencing datasets were compiled from multiple related studies (Table 1), which were downloaded from NCBI SRA and GEO databases (Acland et al., 2014). The raw data contain more than 192 million sequencing reads. These datasets were classified into different clades, species, tissues, and cell lines according to the description on the website or related literature.

**Table 1:** **List of species collected in exo-miRExplorer, and the number of exo-miRNAs in each species**.

**Clade**	**Full**	**Samples**	**exo-miRNA Number**
Mammal	*Homo sapiens*	382	7143
Mammal	*Mus musculus*	128	2574
Mammal	*Sus scrofa*	6	268
Vertebrate	*Gallus gallus*	4	342
Cephalochordata	*Branchiostoma floridae*	1	119
Deuterostome	*Ciona intestinalis*	4	62
Insect	*Bombyx mori*	3	226
Insect	*Drosophila melanogaster*	8	84
Nematode	*Caenorhabditis briggsae*	4	88
Nematode	*Caenorhabditis elegans*	16	549
Nematode	*Caenorhabditis remanei*	4	126
Protozoan parasite	*Trypanosoma brucei*	2	61
Prokaryote	*Escherichia coli*	1	1

### Obtain genome and annotation files

All known miRNAs were downloaded from miRBase (release 21) (Kozomara and Griffiths-Jones, 2014). The genome sequences and transcript sequences were downloaded from NCBI Reference Sequences (RefSeq) (Pruitt et al., 2012), UCSC Bioinformatics websites (Meyer et al., 2013) and other special databases (Table 2). Human (*Homo sapiens*, UCSC hg19), mouse (*Mus musculus*,UCSC mm10), chicken (*Gallus gallus*, v3) and *Ciona intestinalis* (JGI v2.0) genome sequences were downloaded from the UCSC Bioinformatics website; *Caenorhabditis remanei, Caenorhabditis elegans*, and *Caenorhabditis briggsae* genome sequences were downloaded from WormBase(Harris et al., 2014); *Drosophila melanogaster* genome sequences were download from Flybase (Tweedie et al., 2009); *Bombyx mori* genome sequences were download from silkDB (Duan et al., 2010). The *Trypanosoma brucei* genome sequences were download from TriTrypDB (Aslett et al., 2010). Known non-coding RNAs were downloaded from UCSC (Karolchik et al., 2014), Ensembl (Flicek et al., 2014), and Rfam (Gardner et al., 2009).

**Table 2:** **Names of species and databases where the genome sequences were downloaded**.

**Specie**	**Database**	**Edition/Reference**
Human	UCSC Bioinformatics website	UCSC hg19
Mouse		UCSC mm10
Chicken		*Gallus gallus*, v3
*Ciona intestinalis*		JGI v2.0
*Caenorhabditis remanei*	WormBase	Harris et al., 2014
*Caenorhabditis elegans*		
*Caenorhabditis briggsae*		
*Drosophila melanogaster*	Flybase	Tweedie et al., 2009
*Bombyx mori*	silkDB	Duan et al., 2010
*Trypanosoma brucei*	TriTrypDB	Aslett et al., 2010

### Filtering endosome small RNAs derived from host

So far as we know, many studies have used the “map and remove” approach for the identification of exogenous RNAs (Wang et al., 2012; Tang et al., 2013; Pandya et al., 2014; Tosar et al., 2014), and we applied the same strategy with more detailed annotation. We firstly build a reference dataset which contained a variety of host sequences, including genome sequences, mRNAs, tRNAs, rRNAs, snoRNAs, lncRNAs, and microRNA precursors. In this dataset, we collected all possible transcripts. We mapped the reads to genome and then to transcript, in order to solve the cases like small RNAs derived from exon-exon junction region of mRNAs and gene fusion. Small RNA sequencing reads were firstly mapped to these reference sequences with bowtie program (Langmead et al., 2009). If a read can map to any sequence in the reference dataset with one mismatch allowed, it will be filtered as the endosome small RNAs. The left reads are used for exo-miRNAs identification. We firstly classified all the mature microRNAs from miRBase according to their source species and source kingdom. Sequencing reads were exactly aligned to these microRNAs and classified as “exo-miRNAs from the same kingdom” and “exo-miRNAs from another kingdom.” To reduce the false positive as far as possible, exo-miRExplorer provides multiple features of the potential exo-miRNAs, such as the “Supporting number,” which represents the number of particular miRNA in multiple datasets. An RPM (Reads Per Million) value was used to evaluate the expression of exo-miRNAs (Motameny et al., 2010). These features may provide users to gain exo-miRNAs for more evidence. The identified exo-miRNAs were clearly classified, annotated, and loaded into a local database. The microRNA gene names in the exo-miRExplorer follow the naming guidelines described by MicroRNA Registry (Kozomara and Griffiths-Jones, 2014).

### The expression of exo-miRNAs

“Expression” tool was developed to demonstrate the abundance and frequency of “non-metazoa” microRNAs. It will provide a comprehensive view of exo-miRNAs in a particular tissue or cell line. Users can choose their source of interest by clicking the “select” box in the top middle, after which a heat map graph will be displayed on the web page. The horizontal list represents the miRNA family, and the vertical list indicates the exogenous species. Each color grid demonstrates the “frequency value” of the particular exo-miRNA. The “frequency value” of each exo-miRNA is evaluated by a method modified from reads per million (RPM) values (Mortazavi et al., 2008) and calculated by the following formula:

$$F_{ij}=\sum_{k=1}^{n}\frac{S_{ijk}}{T_{k}}*10^{9}$$

where i represents the species; j represents the miRNA family; k represents the source of the experiment, which contain n datasets; S represents the reads number of the exo-miRNA in the kthdataset; T represents all the sequencing reads in the kth dataset. There are 3 ways to show the order of the heatmap graph: (1) by miRNAs, sorting the miRNAs by name on the horizontal axis; (2). by species, ordering the species by name on the vertical axis; (3). by frequency, sorting the values by “frequency value” on both the horizontal and vertical axes. These three ordering can be chosen from the pull-down box on the top right. Changes of the order trigger dynamic effects on the website that provide the user with comprehensive information about exo-miRNAs in whole tissues or cell lines from multiple angles.

## Results

### A comprehensively annotated catalog of exo-miRNAs

Exo-miRExplorer has been designed to focus primarily on the exo-microRNAs presented in the small RNA deep-sequencing datasets. A total of 563 small RNA deep-sequencing datasets were compiled from multiple studies. The experiments were performed by 56 distinct laboratories with multiple sequencing platforms. Thirteen species were selected in exo-miRExplorer (Table 1).

**Figure 1:**
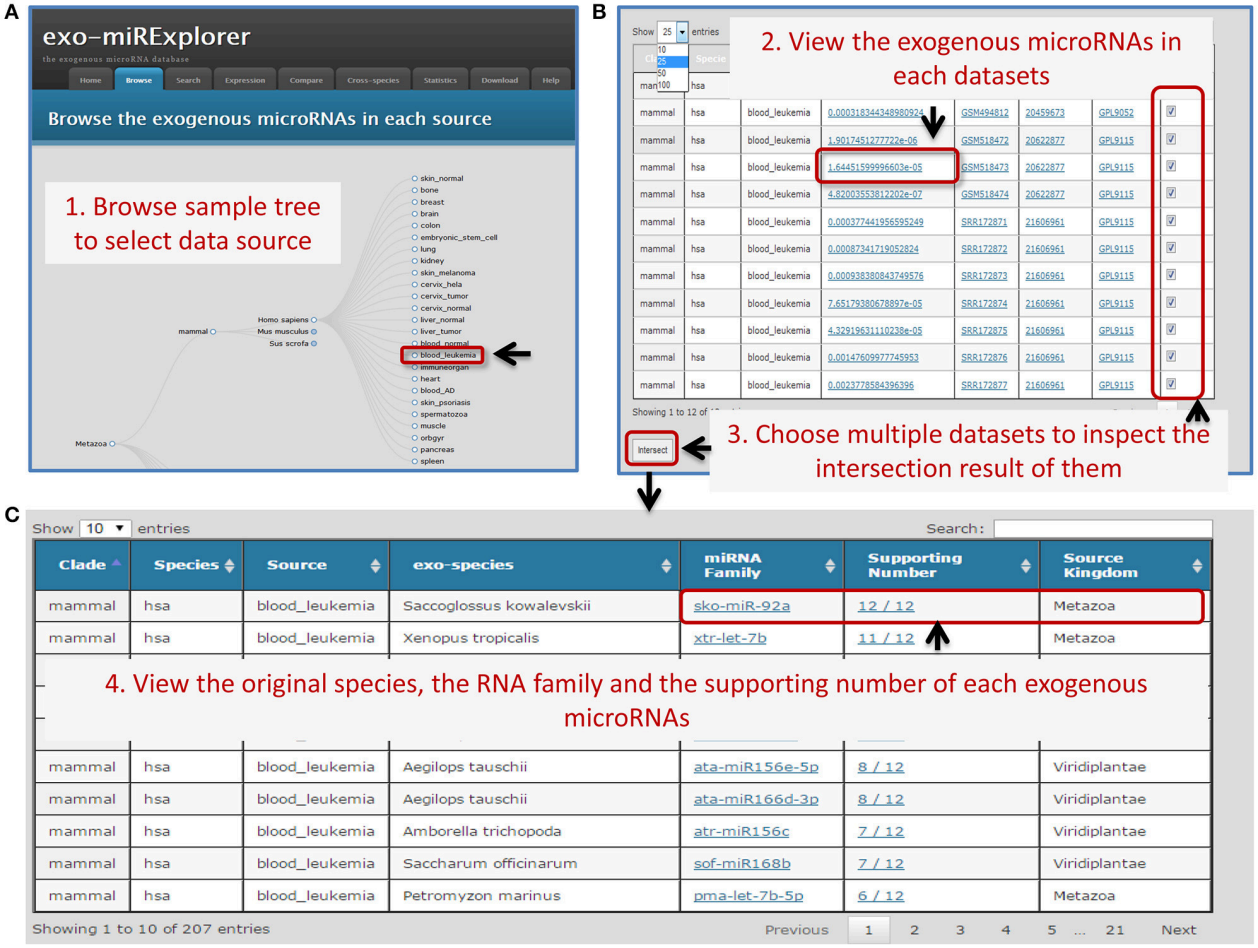
**An Example of the browser interface for retrieving exo-miRNAs. (A)** A screenshot of browse interface. Data is integrated into tree-structured. Users could click the gray circles to unfold the sub-layer, and find the sources of their interest. **(B)** The screenshot of corresponding result of browser. Users can select some of these experiments to see how many times one particular exo-miRNAs appear in these datasets. **(C)** The screenshot of the corresponding summary result. Choosing all 12 collected human leukemia blood samples (“blood_leukemia”) and clicking the “intersect” button, complete information of exo-miRNAs and their occurrence number will be provided. Users can find that sko-miR-92a has the “supporting number” of 12/12, which means all of the 12 samples contain this exo-miRNA.

In exo-miRExplorer, 2754 exo-miRNAs have been clearly annotated and classified according to their original organisms, miRNA family and sequencing source dataset. The sequencing dataset can be viewed from the tree-structure browse page (Figure 1A). Each leaf node represents an experiment sample (tissue, cell line, or development stage). exo-miRNAs in multiple samples can be retrieved by integrated samples (Figure 1). Other features of exo-miRNAs are also listed, including the expression abundance, the source kingdom of species and their portion in the sequencing library. Detailed information about the microRNA, the sample description and the sequencing platform can link to their source annotation website (including miRBase, GEO, and PubMed etc.) by the dynamic hyperlink.

**Figure 2:**
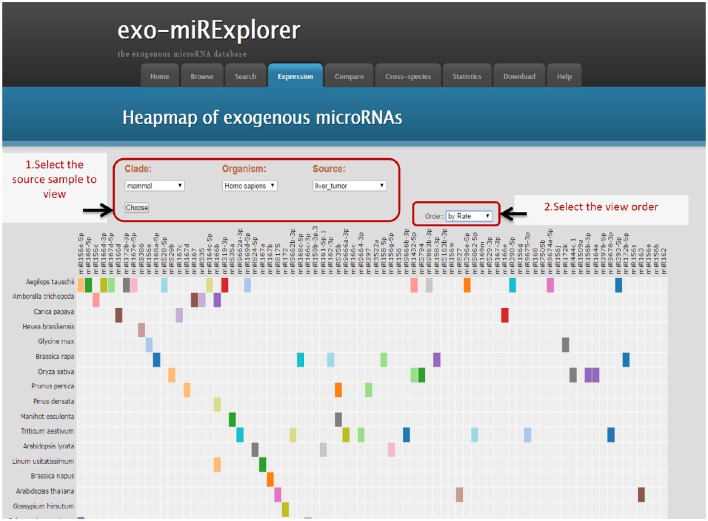
**Illustrative screenshots of the dynamic heatmap browser**. The heatmap browser provides the exogenous species, microRNA families and frequency information of all the exo-miRNAs from a specific source. Users can change the selection box at top-left for clade, organism and special tissues, or cell lines; then, Exo-miRExplorer generates a dynamic heatmap graph. As shown here, most of the grids are gray, which indicates that no miRNAs were detected. The prominent color grid represents the particular microRNA that is detected, and the different color represents the “frequency value” of the miRNAs. Users simply click on the color grid, which links to provide details regarding miRBase to see details of these miRNAs. There are three manners to order these exo-miRNAs: By “frequency value”, by miRNA names, or by species names. Each manner can be chosen by changing the select box at the top-right.

To fully demonstrate the expression profiles of exo-miRNAs, we provide a heat map view of each sample. To fully exclude the endogenous microRNAs (endo-miRNAs), a strict filtering standard was used to get rid of all the metazoa miRNAs (Materials and Methods). In the “Expression” tool, only “non-metazoa” exo-miRNAs are shown in the expression page (Figure 2). Next, we will use exo-miRExplorer to verify if these exo-miRNAs actually present in cells or result from experimental contamination.

### Comparative analysis of exo-miRNAs between “intra-study” and “inter-study”

**Figure 3:**
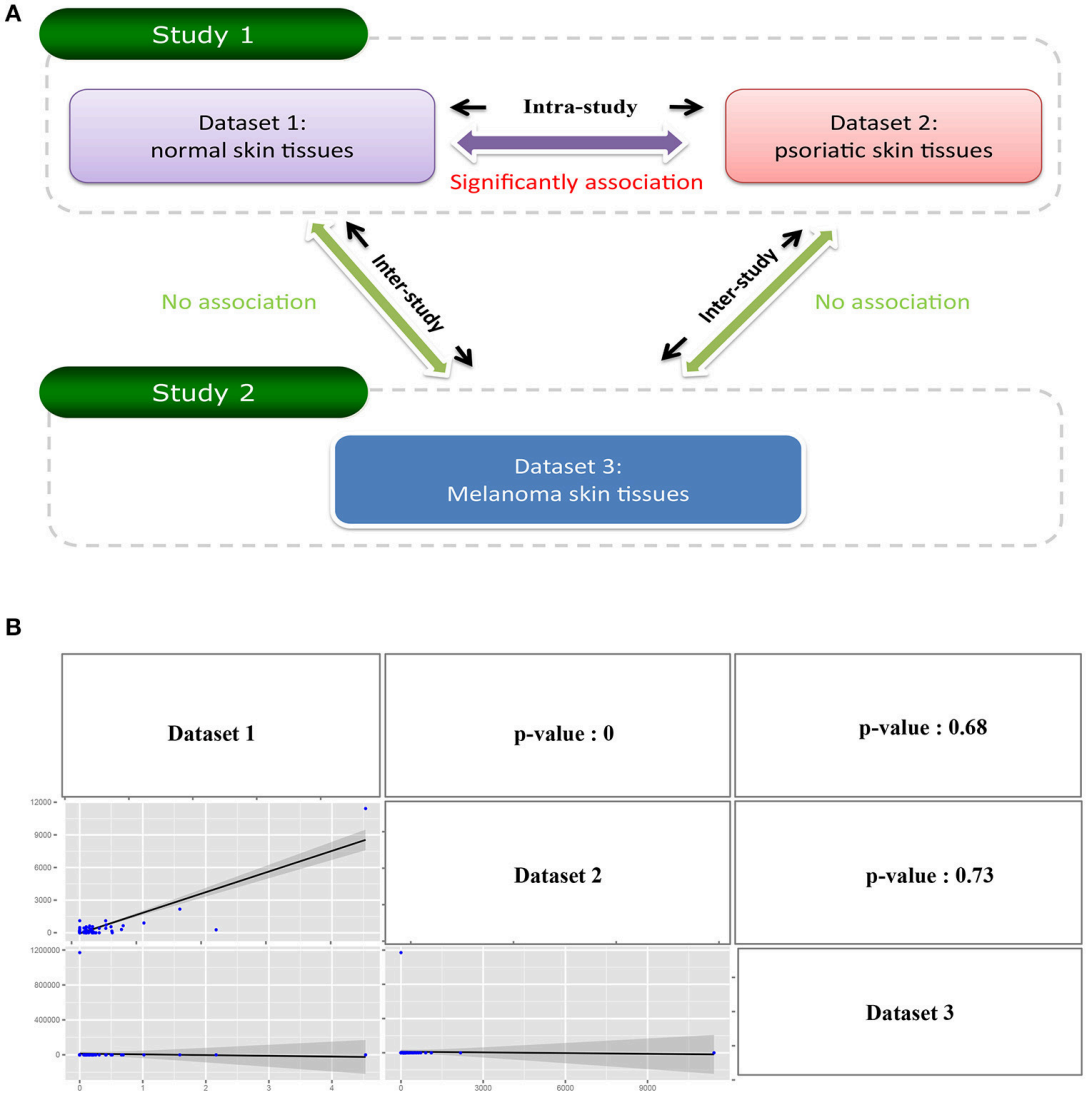
**Illustrate comparative investigation of exo-miRNAs between intra-study and inter-study. (A)** Diagram of the relationship between three datasets. Dataset 1 and Dataset 2 belongs to the same study (study 1), and Dataset 3 belongs to the other study (study 2). The comparison between Dataset 1 and Dataset 2 is “Intra-study” analysis while the comparison between Dataset 1 with Dataset 3 or Dataset 2 with Dataset 3 is “inter-study” analysis. **(B)** The result of the comparative analysis. The figure contains a matrix with nine grids. The strings on diagonal represent the corresponding row names and column names of the matrix. The figures on the lower triangle are the pairwise relations between the row name and column name where they located, and the upper triangle is the corresponding *p*-value of the pairwise comparison.

Exo-miRExplorer provides a “compare” tool for researchers to carry out a comparative analysis of exo-miRNAs in different sequencing datasets. Users would like to find whether there are common exo-miRNAs between different datasets. If one exo-miRNA frequently presents in multiple datasets produced by the same research group (the “intra-study”), it has a high probability to be the pollution during the process of sequencing. On the other hand, if one exo-miRNA frequently presents in the datasets performed by different research groups (“inter-study”), it may be genuine functional exo-miRNA in the cell. Following this idea, we used the “compare” tool to perform the analysis. For “intra-study” analysis, two groups of small RNA sequencing datasets which were performed by the same laboratory we chosen (Joyce et al., 2011). Dataset 1 was from the normal skin tissues, while dataset 2 was from the psoriatic skin tissues (Figure 3A and Figure S1A). The comparison result showed that, in total, there are 275 exo-miRNAs in either of the datasets, 44.36% (122 out of 275) of them were observed in both of the datasets. Pearson correlation analysis revealed a strong positive association between these two datasets (*p*-value < 0.0001) (Figure 3B). We next performed “inter-study” comparison. These datasets were obtained from melanoma skin tissue, but were performed by another laboratory (Figure 3A) (Stark et al., 2010). When comparing “dataset 1” and “dataset 3” (Figure S1B), only 3.2% (6 in 186) of exo-miRNAs was found to be shared between the two groups, and no significant correlation was observed between the two datasets (*p*-value = 0.68); A similar result was observed between the comparation of “dataset 2” and “dataset 3” (*p*-value = 0.73) (Figure 3 and Figure S1C). The comparative analysis results demonstrated a significant correlation of exo-miRNAs between datasets within “intra-study,” but no correlation between “inter-study” which were performed using the same type of tissue (Figure 3A). Combined with previous investigations (Tosar et al., 2014), we can deduce that these “intra-study” shared exo-miRNAs are more likely to derive from contamination. These contaminations may be derived from the preparation of samples in the laboratory or from the sequencing process in the sequencing center.

### Inspect exogenous plant-derived microRNAs in animal samples

**Figure 4:**
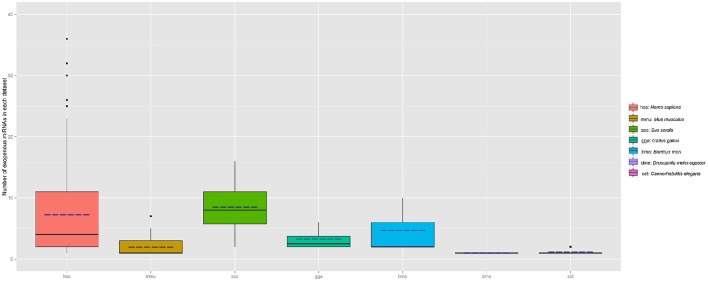
**Distribution of plant miRNAnumber in each species (sample number >3)**. The number of exo-miRNA in each dataset is calculated and grouped by different species. Boxplot is used to illustrate the distribution of exo-miRNA number in each species, the solid line represents the medium number of exo-miRNA in each species, the dotted line represents the mean value of exo-miRNA.

To determine whether the plant miRNAs exist in the samples of animal and other species, all samples collected in exo-miRExplorer were examined to find exo-miRNAs derived from plants. The results showed that plant-derived exo-miRNAs existed in all of the categories in exo-miRExplorer (Figure 4). In human samples, 237 plant-derived miRNAs were detected in 382 sequencing samples (Table S1). These miRNA families include miR168, miR156, miR166, miR167, and miR172 etc.

The most frequently presented miRNA is miR168a, which was detected in 95 samples from 11 distinct studies. The average abundance is 22114.9 RPM. In total, miR168a is presented in 155 samples and multiple species, including human, mouse, chicken, worm, even in the lower eukaryote parasite *T. brucei* and prokaryote *Escherichia coli*. It should be noted that *T. brucei* is an early-branched single-cell animal. It has been believed that microRNA genes are absent from this ancient organism (Wen et al., 2011; Zheng et al., 2013). In addition, prokaryote *E. coli* even lacks the RNAi mechanism, which is replaced by a distinct system called CRISPR-Cas (clustered regularly interspaced short palindromic repeat). This system is used to against invading phages and plasmids (van der Oost et al., 2009). Moreover, both of these two organisms do not rely on a plant as their food and, therefore, there is no chance for plant-derived small RNAs transferred into these two organisms by food-intake.

**Figure 5:**
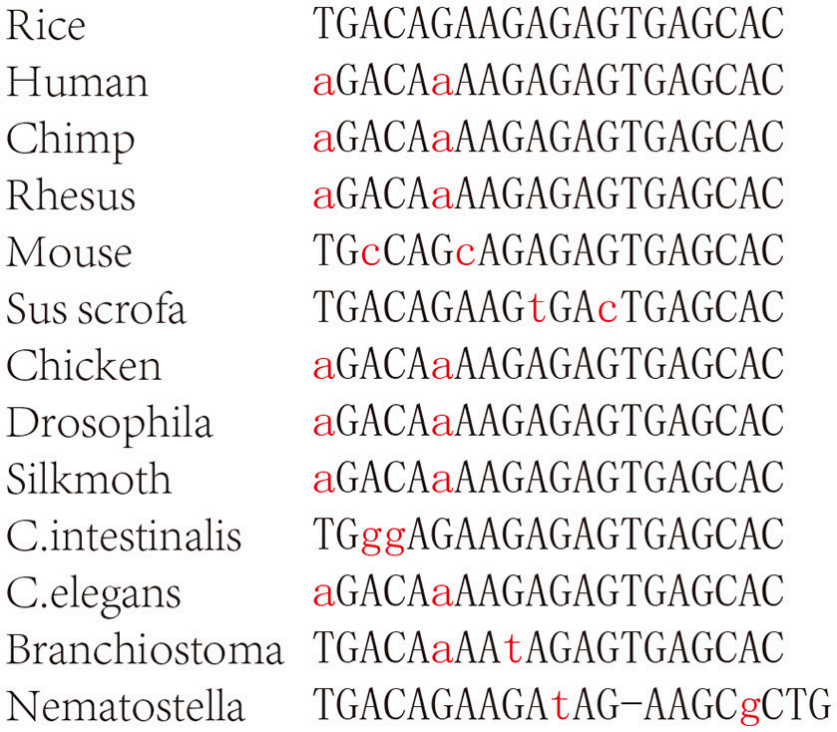
**The similarity sequence of osa-miR156a in other organisms**.

miR156a is another frequently observed exo-miRNA from plants. It could be detected in 65 samples in exo-miRExplorer with the average abundance of 86.69 RPM. miR156 is a conserved family in plants, which has been shown to play important roles in controlling the agronomic traits of plants (Jiao et al., 2010; Miura et al., 2010; Zheng and Qu, 2015). Moreover, a recent investigation found that plant miR156a has significant sequence identity to the microRNA in a representative of Cnidaria (sea anemone Nematostella) (Moran et al., 2014). We then inspected this sequence in other animal species. To our surprise, miR156a has highly sequence similarity to multiple animal sequences, including human, chimp, Rhesus monkey, mouse, pig, chicken, insects, and *Branchiostoma floridae* (Figure 5). In addition, the *Helianthus argophyllus* miR156c, which is in the same family with miR156a, could perfectly match to the human genome in two regions. These two regions are located in the intron region of two distinct genes, anaplastic lymphoma receptor tyrosine kinase (ALK), and autism susceptibility candidate 2 (AUTS2) (Figure S2). Therefore, we consider that it is highly possible that the miR156 sequence found in NGS data is actually derived from mammal itself. Therefore, the high sequence similarity of plant-derived exo-miRNA with the animal reference genome sequence will remind us to take special caution of these exo-miRNAs in our own analysis.

### Investigation of virus-derived MiRNAs in B-cell samples

In addition to contamination, scientists are more concerned about genuine exo-miRNAs in the samples. Recently, several researchers have found viral transcripts in lymphoma cell lines by deep-sequencing technology (Cao et al., 2015). Considering that virus could be transferred into host cells, it is intriguing to assume that exo-miRNAs from the virus could be detected in host cells. We then chose immune organ as an example to investigate all the virus-derived exo-miRNAs.

**Figure 6:**
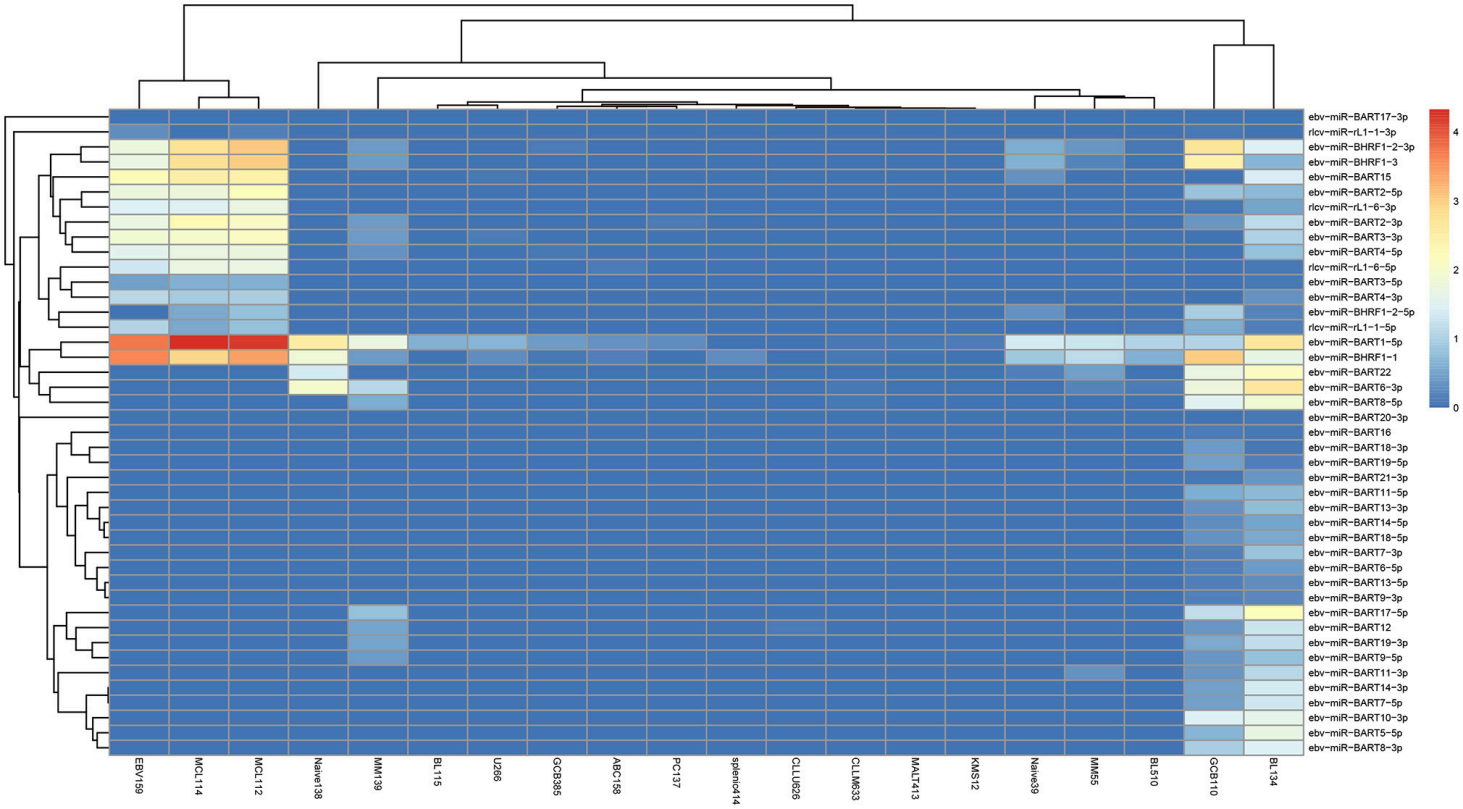
**The expression of virus-derived miRNAs in each B-cell samples**. The horizontal axis illustrates the abbreviation of the cell-line according to the original names in GEO. The vertical axis shows the name of exogenous RNA. The color represents the normalized RPM value of the exo-miRNA.

In exo-miRExplorer, there are 43 samples related to lymphoid cells and bone marrow samples, which were performed by two different groups (Jima et al., 2010; Schotte et al., 2011). In total, 43 virus-derived exo-miRNAs were found (Figure 6). Most of them are derived from epstein-barr virus (EBV) (38 in 43 = 88.37%), a human herpesvirus. Among them, ebv-miR-BART1-5p and ebv-miR-BHRF1-1 are two most frequently observed miRNAs (Table S2). These miRNAs are significantly high expressed in the EBV activated B-cell line (ebv-159) and two mantle cell lymphoma samples (MCL112 and MCL114) (GSM497062 and GSM497061) (Figure 6). However, these EBV-derived miRNAs are rarely observed in other tissues and cell lines. EBV has been demonstrated to be associated with a number of B-cell cancers and lymphoproliferative disorders (Saha and Robertson, 2011). The finding that exo-miRNAs presented in the deep-sequencing results of human samples and cell lines will help to reveal the interaction relationship of viruses and hosts. More experiments should be designed to reveal the potential functions and application value of these virus-derived exo-miRNAs.

## Discussion

**Figure 7:**
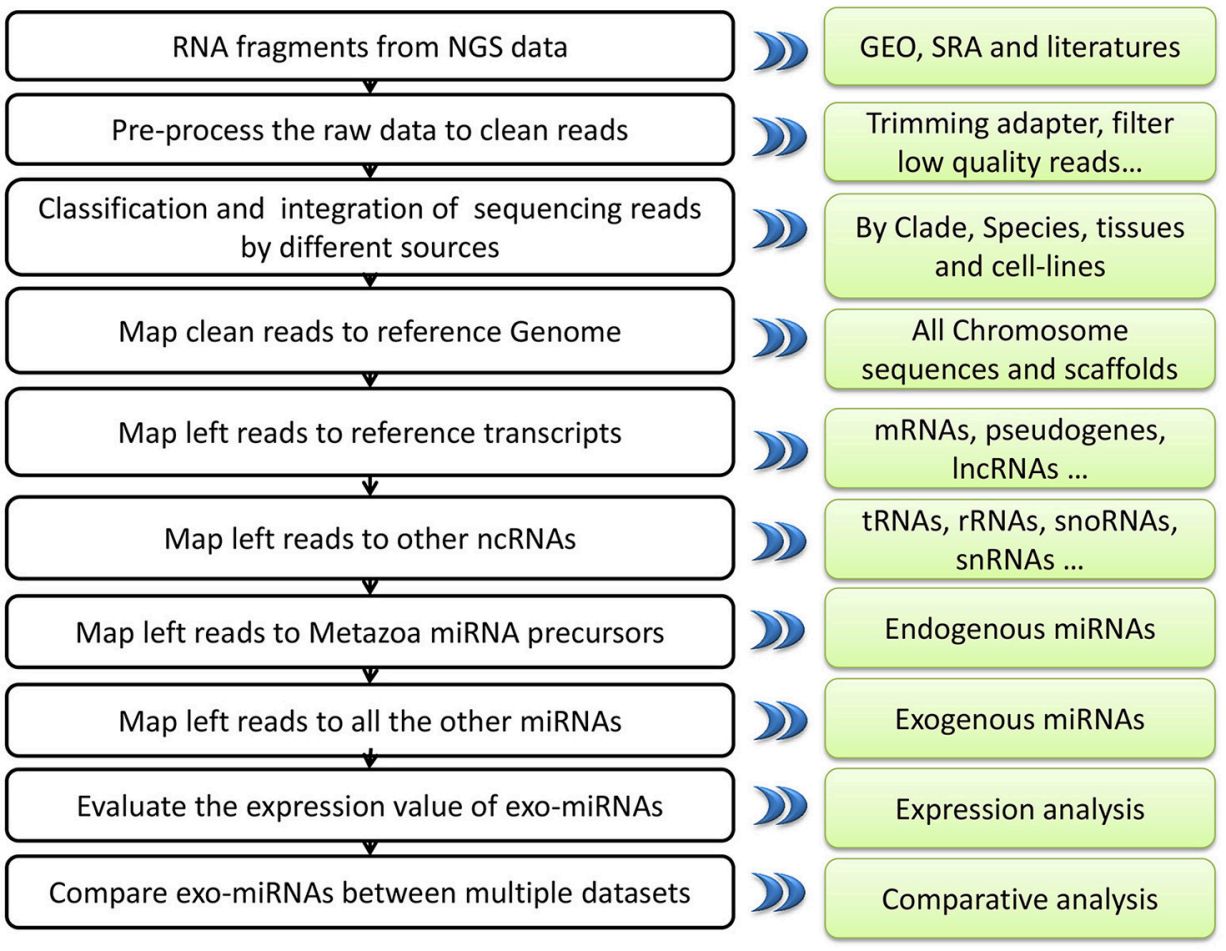
**A systematic overview of EXO-miRExplorer core framework**. All results generated by EXO-miRExplorer are deposited in a MySQL relational database and displayed in the modern web browsers.

Next–generation sequencing technology can deeply detect nearly all of the RNAs in samples. Nevertheless, it can also bring in some pollution fragments to the result (Olarerin-George and Hogenesch, 2015). The major goal of exo-miRexplorer is to help the researchers to identify the exo-miRNAs from small RNA deep sequencing data (Figure 7). These exo-miRNAs might either be common contaminations or transferred from other species. In one hand, if they are frequently observed in exo-miRexplorer, they have a high probability of a common contaminations. exo-miRExplorer has demonstrated 30 most frequently presented exo-miRNAs in statistics page (Figure S3), researchers must be cautious about these exo-miRNAs if they present in their data. These contaminations are quite similar with the endogenous RNAs, it will make researchers overestimate the expression level of endogenous genes, which will possibly lead to bias or even completely wrong conclusions for the experiments. In addition, if most of the sequencing reads are derived from exo-miRNAs, which point out the data cannot be used for further analysis. In another hand, if these exo-miRNAs are transferred from other species, we should find the source of these exo-miRNAs in exo-miRexplorer, and try to reveal the mechanisms of their cross-species regulation. Therefore, it's of great importance to our database that we want to remind the researchers to be careful about the frequent potential exo-miRNAs that might either be common contaminant or new mechanism yet to discover.

When the sequencing reads are mapped to the reference genome, analysis methods always allow 1–2 nt error tolerance. However, our finding revealed that exogenous plant miR156a could match multiple animal genomes with 2 mismatches. miR156a had a relatively high abundance in the sequencing datasets and could be found in many public datasets. If the mapping criterion is restricted to 2 mismatches, these contamination exo-miRNAs will interfere with the measurement of actual gene expression number. Therefore, based on our experience, we strongly suggest that the parameter of mapping sequencing reads to genome should be only one mismatch allowed to exclude the impact of exo-miRNAs. Moreover, these highly similar exo-miRNAs need deeply classification and clearly excluded by special parameters when processing small RNAs sequencing data.

The latest investigations revealed that exogenous RNA from virus or parasites could be detected in the sequencing datasets, and could be used as novel biomarkers for diagnosis of pathogen infection (Li et al., 2010; Hoy et al., 2014; Kawano et al., 2014; Tritten et al., 2014). Interestingly, results revealed that EBV viral microRNAs could induce tumor metastasis in nasopharyngeal carcinoma (Cai et al., 2015). In fact, viral miRNAs are also found in exo-miRExploer at an abundance level. In addition, these viral miRNAs show tissue-specific expression, especially in immune associated organs. In most viral infections, the host innate immune system can block the viral replication cycle. However, whether these viral miRNAs observed in immune organs are the cause or the consequence of the host immune response is unclear. It is necessary therefore to investigate the relationship between viral miRNAs and cancers systematically. Obviously, further works are required to understand how these virus-derived RNAs contribute to the capacity of pathogens in communicated with the host cells. We hope that our results could inspire the creative scientists who are interesting to investigate this fascinating research field.

In our experience, we consider that the most important and difficult thing is to distinguish the contamination exo-miRNAs from the actual infected exo-miRNAs. Here we provide three major features for users to recognize the actual exo-miRNAs from contamination: (1) The observed frequency. If one miRNA could be observed in multiple samples performed by independent groups, it should highly possible be the real exo-miRNA. exo-miRExplorer provide “supporting number” for users to inspect this feature. (2) The evolutionary distance between the species which contain the same type of exo-miRNAs. In our analysis, human and *E. coli* have the same type of plant miRNAs, it is hard to believe that these exo-miRNAs are derived from food. When users find the expression of one particular exo-miRNA, they can retrieve all the species which contain the same exo-miRNA in exo-miRExplorer. (3) Whether other miRNAs from the same species could also be observed in the sample? The ratio of miRNAs observed in other species to the total miRNAs in their original specie. If one species could release miRNA to another species, it should not release only one or two types of microRNAs. Although rice miRNAs are frequently observed in other species, most of them are miR-168a and miR-156a. Of 713 miRNAs recorded in rice, only 6.17% of them could be observed in other species. In comparison, 86.36% of recorded 44 EBV miRNAs could be observed in human samples. We consider that these three features can be used to distinguish the real exo-miRNAs from the potential contamination RNAs.

Considering the false positive in miRNA identification and high homology of miRNAs between different organisms, we cannot completely exclude the potential miRNAs in which some fragments are actually derived from endogenous RNAs, though strict filter standard has been used in our analysis. Other possibilities also exist of course. For instance, if genome-sequencing is incomplete, it will inevitably contain sequencing error, and if the RNAs go through some RNA process events like novel splicing, RNA editing, or gene fusion events, they can result in endogenous fragments exactly the same to exo-miRNAs. exo-miRExplorer will keep updating by improving the algorithms and integrating more sequencing datasets to filter false positive results. We also try to answer the questions regarding the mechanism of exo-miRNAs transfer or even communicate with the host molecules.

## Author contributions

LQ, JY, ZL, and LZ conceived and designed the study. LZ, KD performed the data analysis pipeline. AD, JW collected, classified, and handled the source data. LQ, JY, ZL, JW, AD, KD, and LZ wrote the paper. All authors read and approved the manuscript.

## Funding

This research is supported by the Ministry of Science and Technology of China; National Basic Research Program (No. 2011CB811300) from the National Basic Research program (“973” program) to LQ; and the National Natural Science Foundation of China (No. 31230042, 31472058, 31401975, 31471223, 31370791); Guangzhou science and technology plan projects (201504010022). This research is supported in part by the Guangdong Province Key Laboratory of Computational Science and the Guangdong Province Computational Science Innovative Research Team.

### Conflict of interest statement

The authors declare that the research was conducted in the absence of any commercial or financial relationships that could be construed as a potential conflict of interest.

## Supplementary material

Table S1 **List of plant-derived miRNAs in exo-miRExplorer**.

Table S2 **List of virus-derived miRNAs in exo-miRExplorer**.

Table S3 **List of datasets used for exo-miRExplorer**.

Figure S1 **The manipulating steps and results of comparative analysis between “intra-study” (A)** and “inter-study” **(B,C)**.

Figure S2 **Illustrate the two locations of plant microRNA sequence in the human genome**. The sequence of har-miR156c is “TGACAGAAGAGAGGGAGCA,” which can perfectly match two locations in the human genome at the antisense strand. They are chr2:29,682,257–29,682,275 **(A)** and chr7:69,461,270–69,461,288 **(B)**. This figure illustrates the screen shots from the UCSC Genome Browser of these two regions. The black block represents the location of har-miR156c, and the thin lines represent the intron region of gene according to the annotation from multiple databases, including UCSC genes, RefSeq Genes, Ensembl genes, GECODE, and GenBank. The arrows show the transcribe direction of genes.

Figure S3 **Top 30 exo-miRNAs types and number**.
