# Supplementary figures and images for: Exo-miRExplorer: A Comprehensive Resource for Exploring and Comparatively Analyzing Exogenous MicroRNAs
Source: Front Microbiol. 2017 Feb 1;8:126. doi: 10.3389/fmicb.2017.00126 (PMC5285362; doi:10.3389/fmicb.2017.00126)

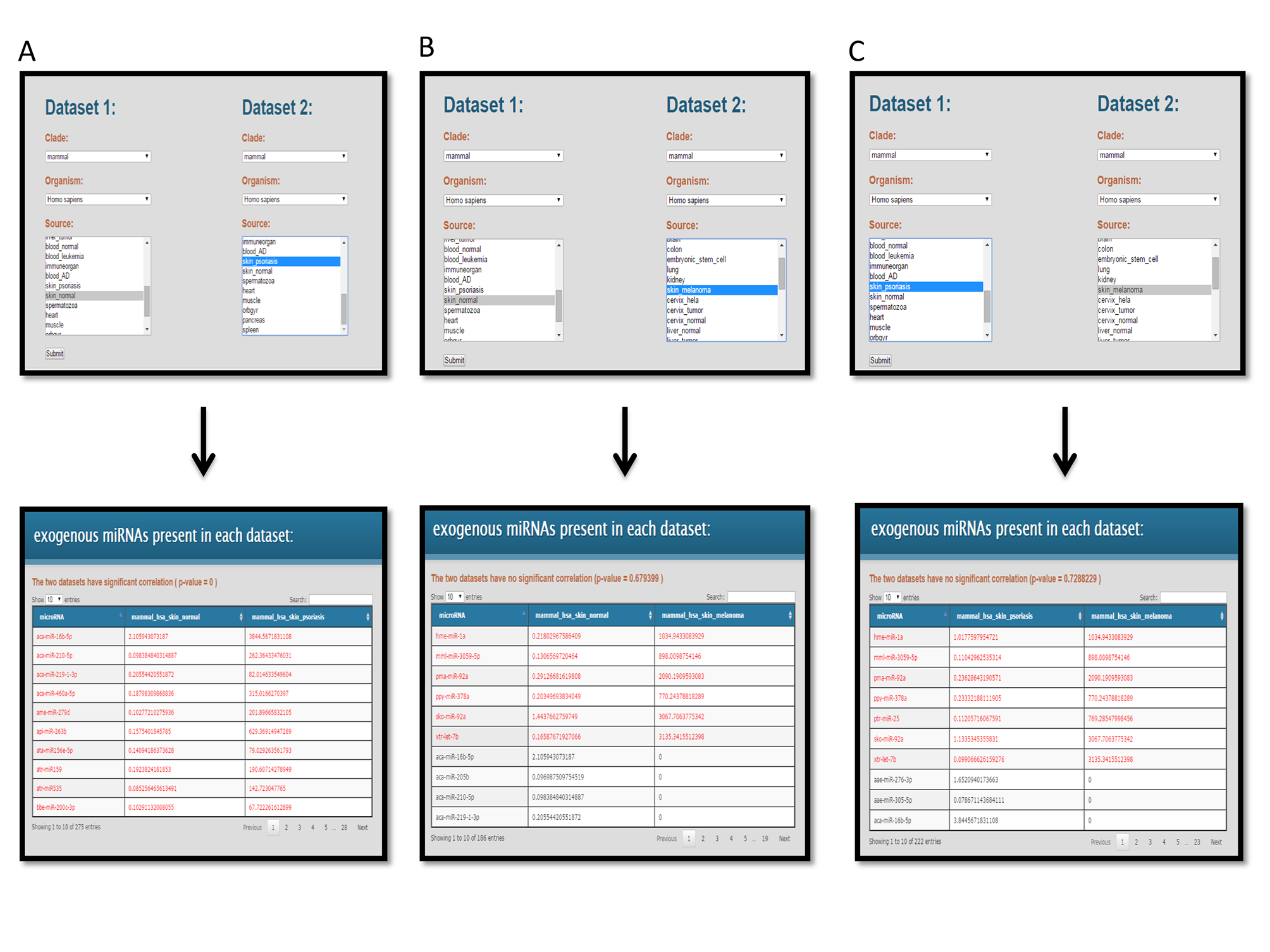

Supplement: Figure S1 — The manipulating steps and results of comparative analysis between “intra-study” (A) and “inter-study” (B,C). [file Image1.TIF]

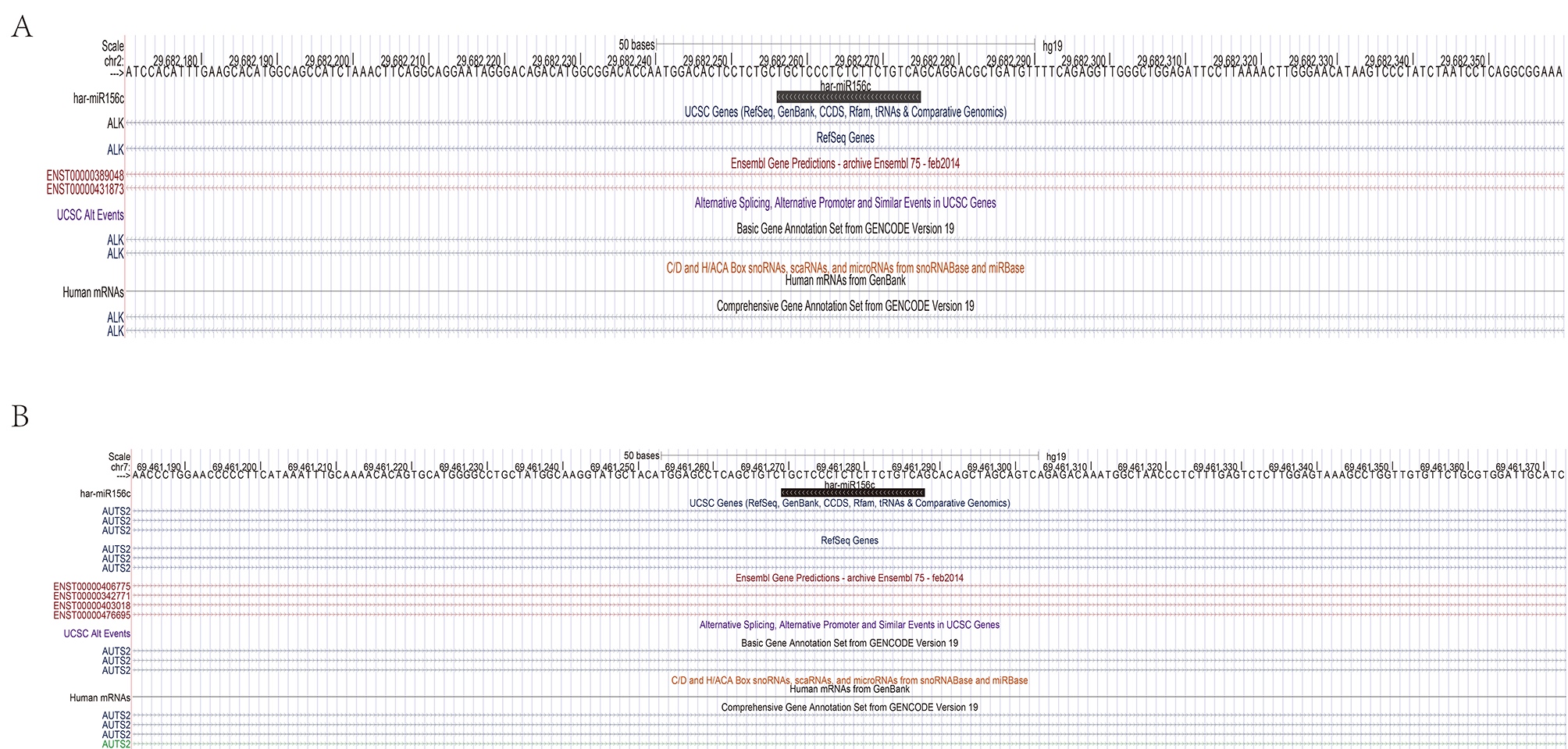

Supplement: Figure S2 — Illustrate the two locations of plant microRNA sequence in the human genome. The sequence of har-miR156c is “TGACAGAAGAGAGGGAGCA,” which can perfectly match two locations in the human genome at the antisense strand. They are chr2:29,682,257–29,682,275 (A) and chr7:69,461,270–69,461,288 (B). This figure illustrates the screen shots from the UCSC Genome Browser of these two regions. The black block represents the location of har-miR156c, and the thin lines represent the intron region of gene according to the annotation from multiple databases, including UCSC genes, RefSeq Genes, Ensembl genes, GECODE, and GenBank. The arrows show the transcribe direction of genes. [file Image2.TIF]

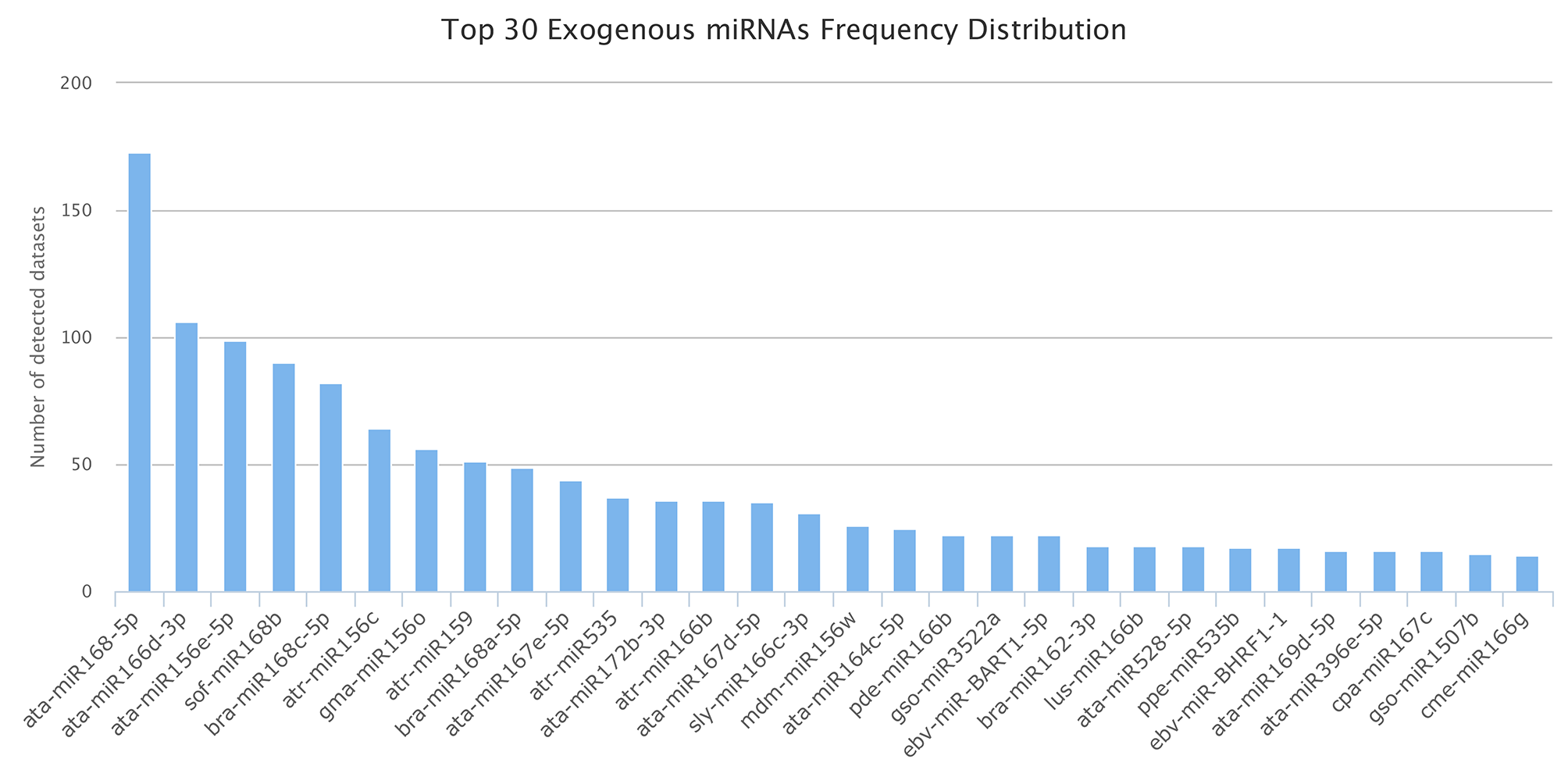

Supplement: Figure S3 — Top 30 exo-miRNAs types and number. [file Image3.TIF]
